# miR-128 Functions as an OncomiR for the Downregulation of HIC1 in Breast Cancer

**DOI:** 10.3389/fphar.2019.01202

**Published:** 2019-10-17

**Authors:** Yan Li, Ying Wang, Xiabo Shen, Xinghua Han

**Affiliations:** ^1^Department of Medical Oncology, The First Affiliated Hospital of USTC, Division of Life Sciences and Medicine, University of Science and Technology of China, Hefei, China; ^2^Department of Epidemiology and Biostatistics, School of Public Health, Anhui Medical University, Hefei, China; ^3^Department of Medical Oncology, Provincial Hospital affiliated to Anhui Medical University, Hefei, China

**Keywords:** breast cancer, HIC1, microRNA, proliferation, apoptosis, invasion

## Abstract

Hypermethylated in cancer 1 (HIC1) is continually decreased in breast cancer. However, the underlying molecular basis of the upstream regulation of HIC1 remains elusive. Here, we showed that HIC1 was downregulated in breast cancer tissues. Bioinformatics analysis identified that miR-128 might potentially target HIC1. HIC1 was proved as the target gene of miR-128 by overexpressing or knocking down miR-128. Additionally, we observed that HIC1 suppression by miR-128 increased cell invasion, proliferation, and reduced apoptosis. Lastly, we found that miR-128 accelerated tumor growth in xenograft mice by inhibiting HIC1. Altogether, this study presents the first evidence that miR-128 suppresses the expression of HIC1 to accelerate breast cancerogenesis.

## Introduction

Breast cancer is one of the most common type of cancer worldwide and the chief cause of death in women (Torre et al., 2015). Although the advance of diagnosis and therapy of breast cancer has significantly reduced the incidence rates and death rates, it still makes up 25% of all cancer cases and 15% of all cancer deaths among females, and the tumorigenesis and progression of breast cancer remain elusive (DeSantis et al., 2011). Therefore, it is of great urgent to study the molecular mechanism of breast cancer and develop new therapeutic agents.

With improved technology and better understanding of tumor biology, more and more genes related to tumorigenesis have been found. These genes provide new targets for tumor treatment (Luo et al., 2009). One of them is hypermethylated in cancer 1 (HIC1), which encodes a transcriptional repressor with plentiful partners and targets and is involved in many cancer processes, such as cell survival, growth, and motility (Pinte et al., 2004; Chen et al., 2005). HIC1 is continually silenced in human cancers, including breast cancer, prostate cancer, colorectal cancer, liver cancer, and lung cancer (Morton et al., 1996; Chen et al., 2005; Jin et al., 2017; Wang et al., 2017), and this is assumed to be ascribed to promoter hypermethylation (Zhang et al., 2010). However, the hypermethylation of HIC1 promoter is detected not only in tumors but also in normal breast ductal (Zhang et al., 2010), prostate epithelium tissues (Morton et al., 1996), and brain (Rood et al., 2002). These findings indicate that a previously uncharacterized regulatory mechanism rather than hypermethylation is involved in HIC1 repression.

A class of non-coding, single-stranded small RNAs named microRNAs (miRNAs) has emerged as a major regulator of tumorigenesis in the last few years, including breast cancer (Iorio et al., 2005; Garzon et al., 2009; O’Day and Lal, 2010). miRNAs bind the complementary sites in the 3’-untranslated regions (3’-UTRs) of target mRNAs, post-transcriptionally inhibiting target gene expression (Bartel, 2009). miRNAs regulate various biological processes through this mechanism, such as cell proliferation, differentiation, apoptosis, migration, and metabolism (Krol et al., 2010). miR-128 is one of the miRNAs frequently increased in several types of cancers (Katada et al., 2009; Pang et al., 2009; Cai et al., 2017), suggesting that miR-128 serves as an oncomiR to promote cancer cell progression. However, the precise expression pattern and underlying molecular basis of function of miR-128 on the development and progression of breast cancer remain to be fully elucidated.

In this paper, we showed that miR-128 ectopic increased in human breast cancer tumors and can target a tumor suppressor gene named HIC1, resulting in the downregulation of HIC1 protein level and consequently promotes proliferation and invasion and inhibits apoptosis of breast cancer cells.

## Materials and Methods

### Cells and Clinical Patient Tissues

MDA-MB-231, T-47D, and MCF-7 cell lines were purchased from the Shanghai Institute of Cell Biology, Chinese Academy of Sciences (Shanghai, China). MCF-7 was cultured in Dulbecco's Modified Eagle Medium (DMEM), T-47D was cultured in 1640, and MDA-MB-231 was cultured in L15 supplemented with 10% (v/v) fetal bovine serum (FBS) (GIBCO, CA, USA) at 37°C in a 5% CO2 and water-saturated atmosphere. Fourteen paired breast cancer and normal adjacent tissues were obtained from the First Affiliated Hospital of USTC, and the clinical features of the patients are listed in **Supplementary Table 1**. All samples involved were approved by the ethics committee and Institutional Review Board of the First Affiliated Hospital of USTC and the written informed consent from each patient.

### RNA Extraction and Quantitative RT-PCR

Total RNA was isolated from patient tissues and cells using TRIzol Reagent (Invitrogen, Carlsbad, CA) according to the manufacturer’s instructions. Stem-loop qRT-PCR assays using TaqMan miRNA probes (Applied Biosystems) were performed to analyze the levels of miRNAs as described previously (Jin et al., 2017). Briefly, real-time PCR was performed using a TaqMan PCR kit on an Applied Biosystems 7300 Sequence Detection System (Applied Biosystems). The reactions were incubated in a 96-well optical plate at 95°C for 10 min, followed by 40 cycles of 95°C for 15 s and 60°C for 1 min. After the reaction, the cycle threshold (CT) data were determined using fixed threshold settings, and the mean CT of the triplicate PCRs was determined. A comparative CT method was used to compare each condition to the controls. The relative levels of the miRNAs in cells and tissues were normalized to U6. The amount of miRNA relative to the internal control U6 was calculated using the 2−∆∆CT equation, in which ∆∆CT = (CT miRNA − CT U6)target − (CT miRNA − CT U6) control.

To quantify HIC1 mRNA, RNA was reverse transcribed into first-strand complementary DNA (cDNA) using oligo dT and Thermoscript (TaKaRa). The primers for HIC1 and GAPDH were as follows: HIC1 (sense): 5’-GTCGTGCGACAAGAGCTACAA-3’; HIC1 (antisense): 5’-CGTTGCTGTGCGAACTTGC-3’; GAPDH(sense): 5’-GATATTGTTGCCATCAATGAC-3’; and GAPDH (antisense): 5’-TTGATTTTGGAGGGATCTCG-3’. GAPDH serves as the internal control.

### miRNA Overexpression or Knockdown

miRNA overexpression or knockdown was achieved by transfecting cells with a miRNA mimic or inhibitor. Synthetic RNA molecules, including pre-miR-128, anti-miR-128, and scrambled positive control RNA (pre-miR-control and anti-miR-control), were purchased from GenePharma (Shanghai, China). MCF-7/MDA-231 cells were seeded in 6-well plates and transfected with Lipofectamine 2000 (Invitrogen) on the following day when the cells were approximately 70% confluent. For overexpression of miRNAs, 100 pmol of pre-miR-128 were used. For knockdown of miRNAs, 100 pmol of anti-miR-128 were used. After 6 h, the medium was changed to DMEM/L15 that was supplemented with 2% FBS.

A mammalian expression plasmid (pReceiver-M02-HIC1) was purchased from GeneCopoeia (Germantown, MD, USA). The small interfering RNA (siRNA) (sequence: CCUAGUCUCCUCUAUCGCUTT) targeting human HIC1 was synthesized by GenePharma (Shanghai, China). Total RNA or protein was isolated 24 or 48 h after the transfection.

### Western Blotting

The protein levels were analyzed *via* Western blot using the corresponding antibodies. The protein levels were normalized by probing the same blots with a GAPDH antibody. The antibodies anti-HIC-1 (H-6) (Santa Cruz, sc-271499, Santa Cruz, CA, USA) and anti-GAPDH (Santa Cruz) were used. ImageJ software was used to analyze the protein bands.

### Luciferase Reporter Assay

To test the direct binding of miR-128 to the target gene HIC1, a luciferase reporter assay was performed. PGL3 plasmid encoding a luciferase report gene was purchased from Ambion. Recombinant plasmid of PGL3-HIC1-3’-UTR or corresponding mutant was constructed in our lab. 293-T cells were cultured in 24-well plates, and each well was transfected with 0.1 µg of firefly luciferase reporter plasmid, 0.1 µg of a β-galactosidase (β-gal) expression plasmid (Ambion), and equal amounts (100 pmol) of pre-miR-128 or the scrambled positive control RNA using Lipofectamine 2000 (Invitrogen). The β-gal plasmid was used as a transfection control. Twenty-four hours after transfection, the cells were assayed using a luciferase assay kit (Promega, Madison, WI, USA).

### CCK-8 Assay

MCF-7 cells (1 × 10^4^ cells per well) were seeded into 96-well plates. The cells were assayed at 12, 24, 36, 48, and 60 h after the different transfections using Cell Counting Kit-8 solution (CK04-500, Dojindo).

### Cell Invasion Assay

The invasion ability of MCF-7 cells was tested in a Transwell Boyden Chamber (6.5-mm, Costar) as described previously (Wang et al., 2017). The polycarbonate membranes (8-μm pore size) on the bottom of the upper compartment of the Transwells were coated with 1% human fibronectin (R&D Systems 1918-FN, USA). The cells were harvested 12 h after transfection and suspended in FBS-free DMEM culture medium. Then, cells were added to the upper chamber (2 × 10^4^ cells/well). At the same time, 0.5 mL of DMEM containing 10% FBS was added to the lower compartment, and the Transwell-containing plates were incubated for 6 h in a 5% CO_2_ atmosphere saturated with H_2_O.

### Apoptosis Assays

The apoptosis of breast cancer cells with different transfections was tested using an Annexin V-FITC/PI staining kit (BD Biosciences, CA, USA). The apoptosis assays were performed as previously described (Liang et al., 2015).

### *In Vivo* Studies

All animal experiments were performed according to protocols approved by the National Institutes of Health’s Guide for the Care and Use of Laboratory Animals and were approved by the First Affiliated Hospital of USTC. MCF-7 cells or MCF-7 cells overexpressing miR-128 or HIC1 overexpressing plasmid were injected subcutaneously into 6-week-old female SCID mice (1 × 10^6^ cells per mouse, five mice per group). Mice were sacrificed after 3 weeks, and tumor weights were determined and then further processed for immunohistochemical staining for HIC1 and Ki-67.

### Statistical Analysis

All analyses were performed using GraphPad Prism v.7.0. Error bars shown in graphical data represent mean ± SE of at least three independent experiments. A value of *P* < 0.05 were considered statistically significant using Student’s t -test.

## Results

### HIC1 Deregulation in Breast Cancer

**Figure 1:**
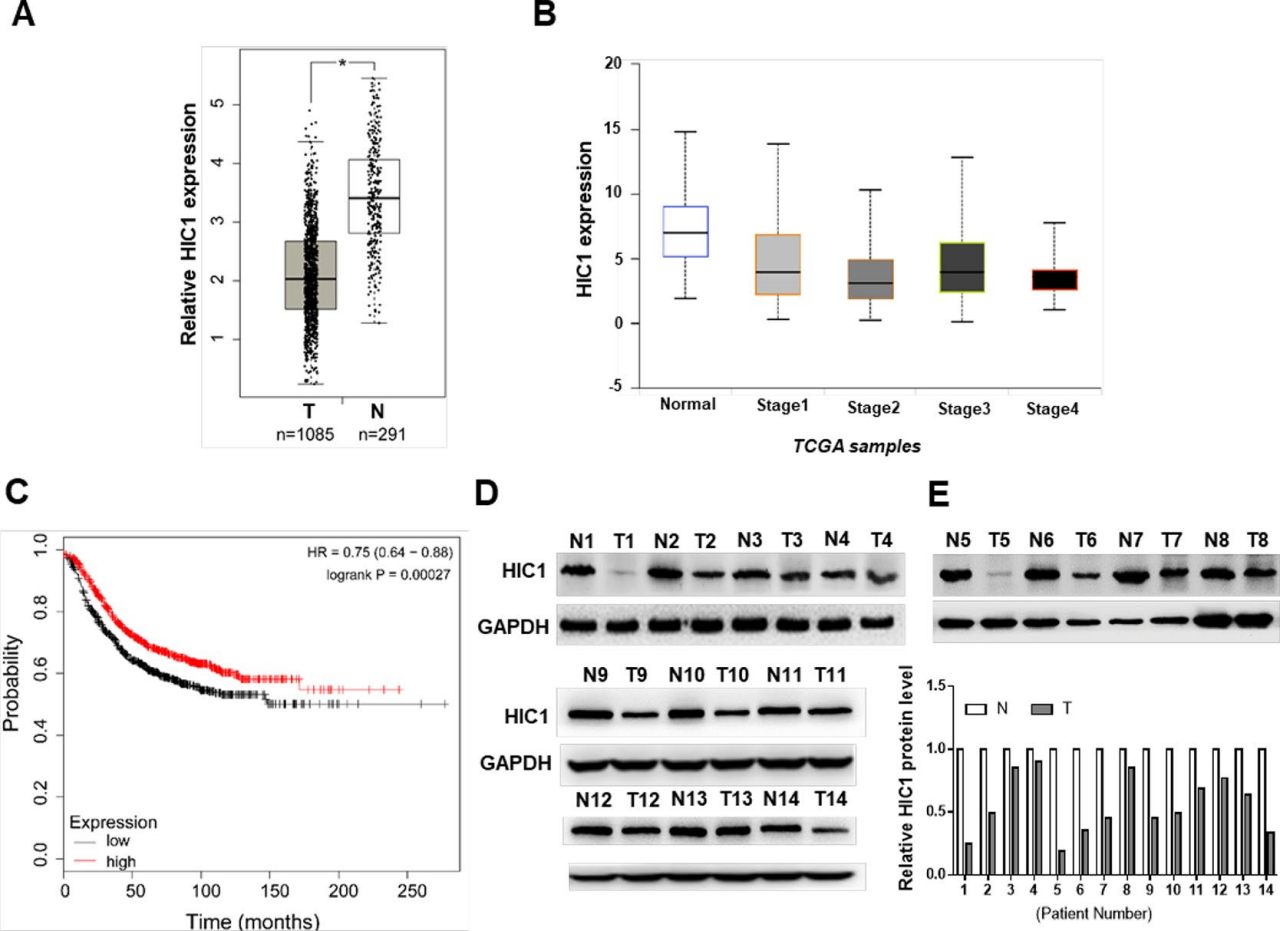
Analysis and measurement of HIC1 expression profiles in breast cancer. **(A)** HIC1 expression in breast carcinoma by TCGA database analysis. **(B)** HIC1 expression was correlated with breast cancer. **(C)** Kaplan–Meier analysis of breast cancer patients’ survival. **(D)** Western blotting analysis of HIC1 protein level in 14 pairs of breast cancer clinical samples. **(E)** Quantitative analysis of HIC1 protein levels in eight pairs of breast cancer tissues. N: noncancerous tissue; T: breast cancer tissue. The results are presented as the mean ± S.E. of three independent experiments. *P < 0.05.

First, we analyzed the patient samples in TCGA database, the results discovered that HIC1 was significantly down-expressed in breast cancers (**Figures 1A, B**), suggesting that the malignancy of breast cancer was related to HIC1 abnormal expression. Patients with low levels of HIC1 showed shorter overall survival time than those with high HIC1 levels (**Figure 1C**). Subsequently, 14 pairs of clinical breast cancer samples were collected to detect the HIC1 expression level by immunoblotting. The results found that the HIC1 protein levels were significantly decreased in the breast cancer tissues (**Figures 1D, E**). Such finding might imply that HIC1 indeed served as a tumor suppressor in breast cancer.

### HIC1 Suppressed Proliferation, Invasion and Enhanced Apoptosis in Breast Cancer Cells

**Figure 2:**
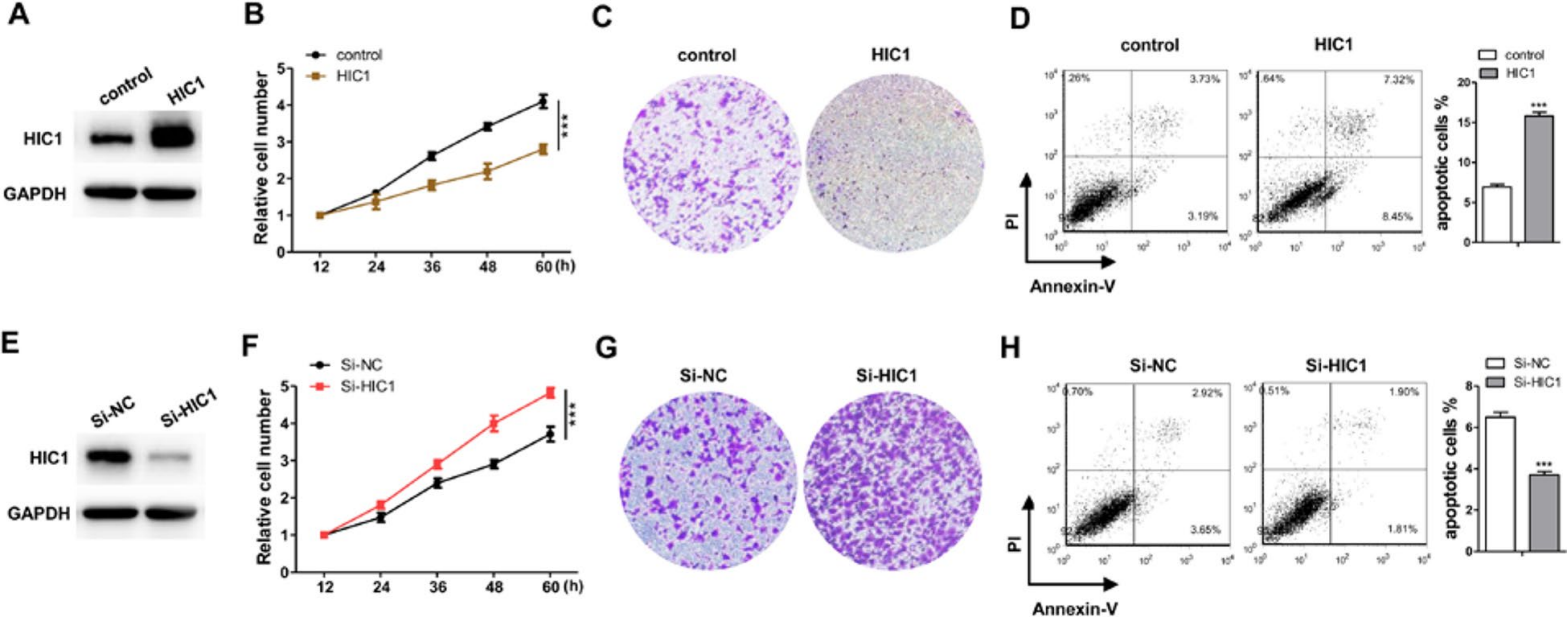
Confirm the function of HIC1 in breast cancer cells. **(A)** Western blot analysis of HIC1 level after transfecting with HIC1 overexpression vector in MCF-7. **(B)** HIC1 overexpression in MCF-7 inhibited cell proliferation. **(C)** HIC1 overexpression in MCF-7 inhibited cell invasion. **(D)** HIC1 overexpression in MCF-7 promoted cell apoptosis. **(E)** Western blot analysis of HIC1 level after the transfection of HIC1 siRNA in MCF-7. **(F)** Knockdown HIC1 in MCF-7 promoted cell proliferation. **(G)** Knockdown HIC1 in MCF-7 promoted cell invasion. **(H)** Knockdown HIC1 in MCF-7 inhibited cell apoptosis. The results are presented as the mean ± S.E. of three independent experiments. ***P < 0.001.

The profile of HIC1 in human breast cancer suggested that HIC1 might act as cancer suppressor (Morton et al., 1996; Chen et al., 2005; Jin et al., 2017; Wang et al., 2017). If so, it must be involved in apoptosis, proliferation, invasion, or other physiological processes in cancer. Therefore, this study mainly focused the effects of HIC1 on cell proliferation, invasion, and apoptosis. First, we tried to change HIC1 expression levels to estimate the role of HIC1 in cancer cell growth. The efficiencies of HIC1 overexpressing plasmid or siRNAs were validated in MCF-7 cells by immunoblotting analysis (**Figures 2A, E** and **Supplemental Figure 1**). The results indicated that cells with a higher HIC1 level were associated with lower proliferation and invasion rates (**Figures 2B, C**) but a faster apoptosis rate (**Figure 2D**). Analogously, cells transfected with HIC1 siRNA grew and invade at higher rates (**Figures 2F, G**) and had a slower apoptosis rate (**Figure 2H**). These results might prove that HIC1 could affect the proliferation and apoptosis of breast cancer cells.

### miR-128 Directly Target the 3’-UTR of HIC1

**Figure 3:**
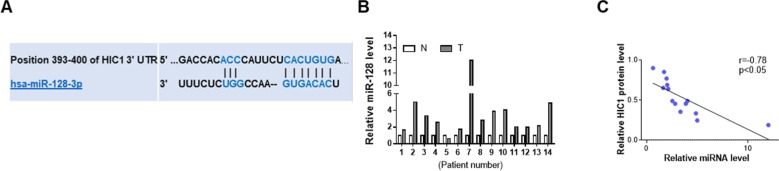
Prediction of HIC1 as target of miR-128. **(A)** Schematic descriptions of the interactions between miR-128 and HIC1 3’UTR. **(B)** Quantitative analysis of the relative expression of miR-128 in 14 pairs of breast cancer tissues. N: noncancerous tissue; T: breast cancer tissue. **(C)** Pearson’s correlation scatter plots illustrating the inverse correlation between miR-128 and HIC1 protein levels.

HIC1 had been previously found to exert important influence on cancer (Chen et al., 2005; Uehara et al., 2014), so there might be a certain regulatory mechanism in the upstream of HIC1. The first candidate regulator was miRNAs, which could play a wide role in animal. The possibility of interaction between HIC1 and miRNA was predicted using TargetScan. Finally, miR-128 was selected as a potential regulator of HIC1 after comprehensive consideration. The predicted interaction between miR-128 and HIC1 3’-UTR is shown in **Figure 3A**. Because miRNAs generally showed opposite expression patterns with their targets, we then studied whether miR-128 was inversely correlated with HIC1 in breast cancer. After measuring miR-128 levels in the same 14 pairs of breast cancer tissues and noncancerous tissues, we found that miR-128 expression levels were increased in most of the breast cancer tissues (**Figure 3B**). The inverse correlation between miR-128 and HIC1 protein levels (**Figure 3C**) was further illustrated using Pearson’s correlation scatter plots. Thus, HIC1 was denoted as a potential direct target of miR-128.

**Figure 4:**
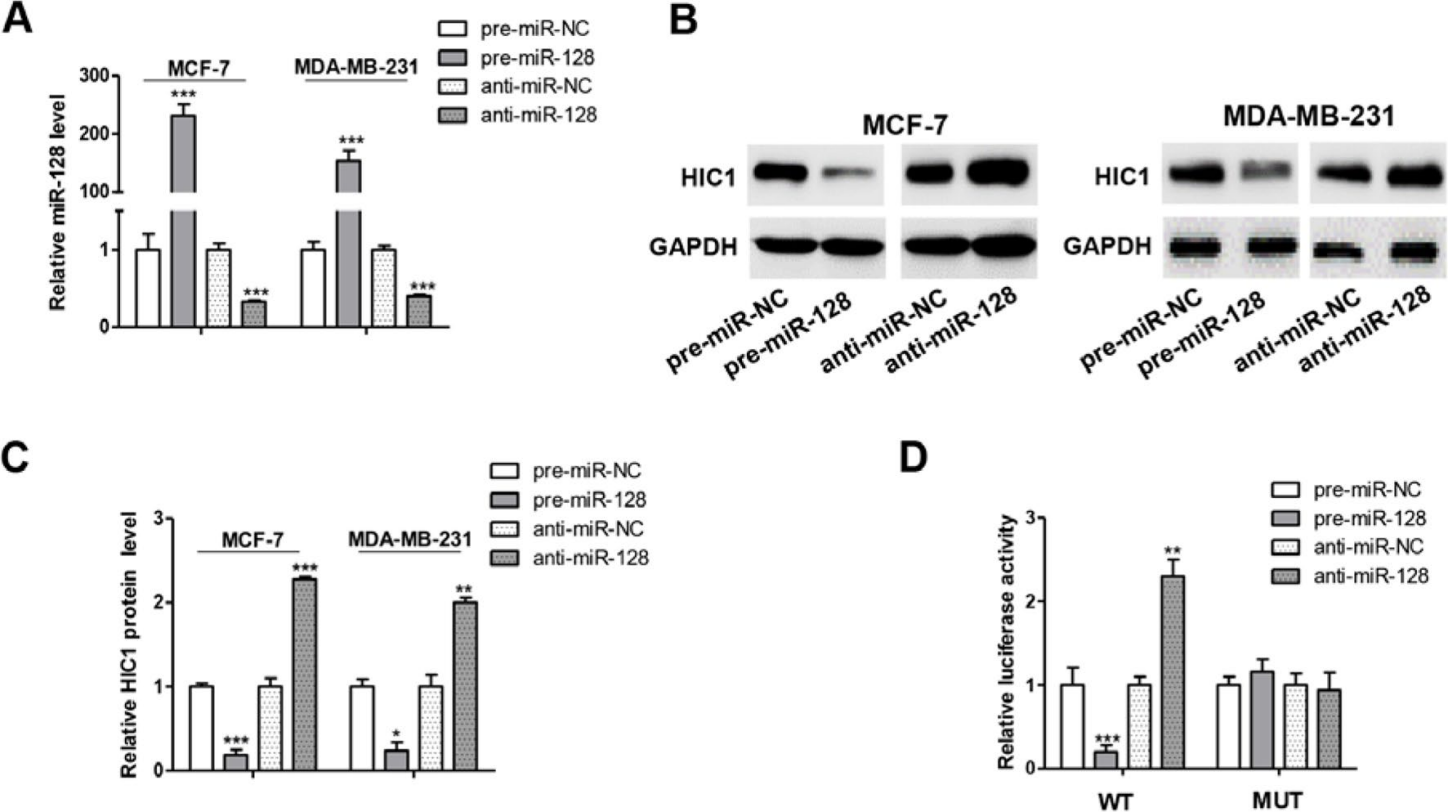
miR-128 directly targets HIC1 3’UTR. **(A)** Quantitative RT-PCR analysis of miR-128 levels in MCF-7 and MDA-MB-231 cells transfected with miR-128 mimics (pre-miR-128), miR-128 inhibitors (anti-miR-128), or scrambled negative control RNA (pre-miR-NC or anti-miR-NC). **(B** and **C)** Western blotting analysis of HIC1 protein levels in MCF-7 and MDA-MB-231 transfected with miR-128 mimic, miR-128 inhibitor, or scrambled negative control RNA. E: representative image; F: quantitative analysis. **(D)** Direct recognition of the HIC1 3’UTR by miR-128. The results are presented as the mean ± S.E. of three independent experiments. *P < 0.05; **P < 0.01; ***P < 0.001.

To further examine the correlation between miR-128 and HIC1, we measured HIC1 protein levels in MCF-7 after transfecting with miRNA mimics or inhibitors. As anticipated, miR-128 was markedly increased when transfected with mimics and decreased dramatically when treated with inhibitors (**Figure 4A**). Consequently, the expression of HIC1 was markedly inhibited by overexpressing miR-128, while miR-128 inhibition markedly upregulated the HIC1 protein levels in breast cancer cells (**Figures 4B, C**). In addition, the above experiments were also repeated in MDA-MB-231 and T-47D (ER positive) cell line, and we observed consistent results (**Figures 4B, C** and **Supplemental Figures 3A, B**).

A luciferase reporter assay was performed to further confirm the direct interaction between 3’-UTR of HIC1 mRNA and miR-128. The full length 3’-UTR of HIC1 mRNA was inserted into the downstream of the firefly luciferase gene in a reporter plasmid. As expected, the luciferase activity was significantly decreased in cells transfected with luciferase reporter plasmid and miR-128 mimics (**Figure 4D**). Specifically, the luciferase reporter activity was markedly increased when miR-128 level was decreased (**Figure 4D**). Moreover, we introduced point mutations (mutation sequence in HIC1 3’-UTR: CACUGUG to GUGACAC), and the mutated luciferase reporter plasmid was unaffected by overexpressing miR-128 (**Figure 4D**), suggesting that the binding sites indeed contribute to the interaction of miR-128 and HIC1. Altogether, our results demonstrate that miR-128 directly bind to the 3’-UTR of the HIC1 mRNA transcript and suppress HIC1 translation.

### miR-128 Promotes Proliferation, Invasion and Inhibits Apoptosis Through Targeting HIC1 in Breast Cancer Cells

**Figure 5:**
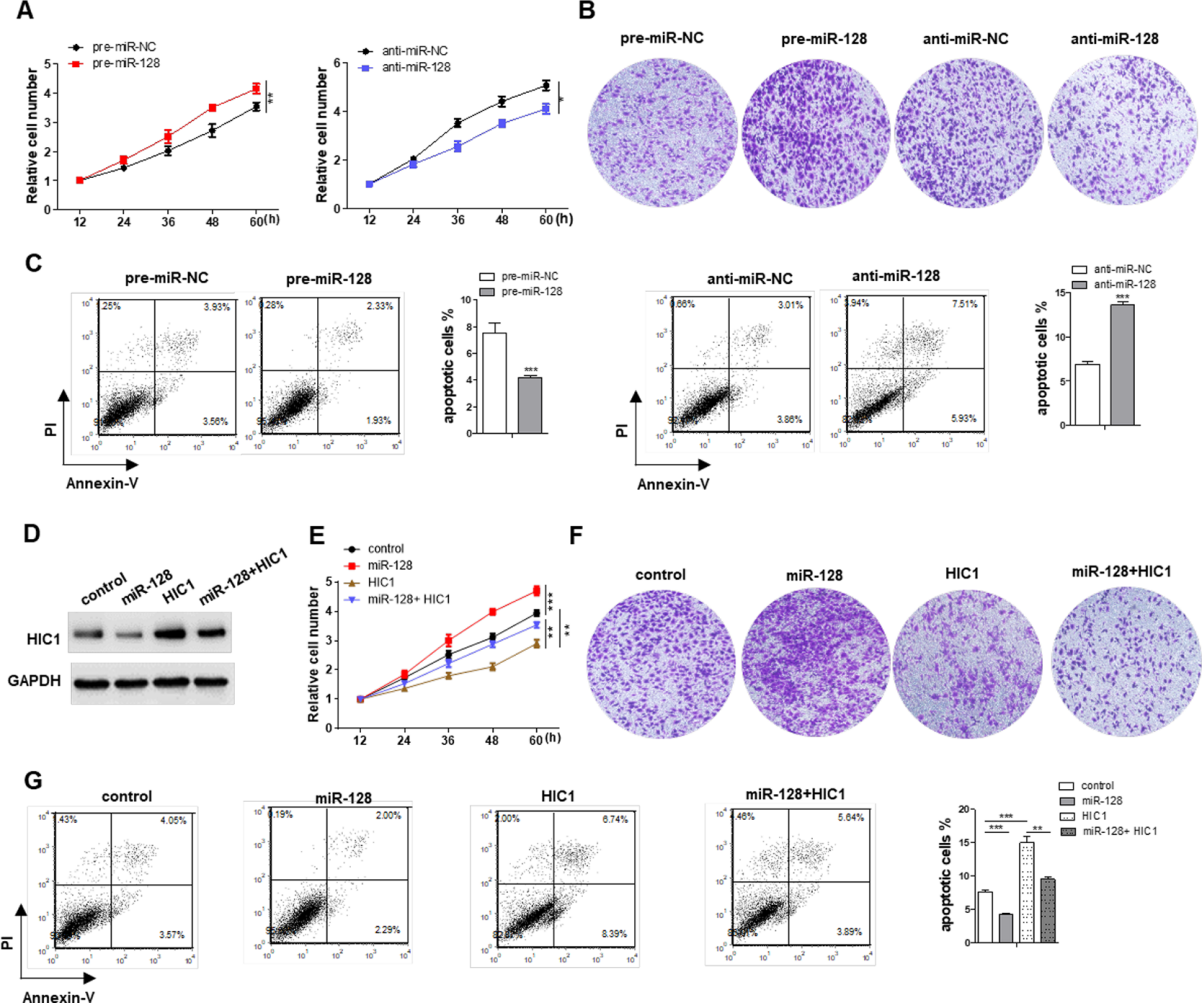
Effects of miR-128-HIC1 axis on proliferation, invasion, and apoptosis of breast cancer cells. **(A** and **B)** miR-128 promoted cell proliferation and invasion in MCF-7, whereas miR-128 inhibitor inhibited cell proliferation and invasion in MCF-7. **(C)** miR-128 inhibited breast cancer cell apoptosis, whereas miR-128 inhibitor promoted cell apoptosis. **(D)** Western blotting analysis to measure HIC1 protein levels in MCF-7 transfected with scrambled negative control RNA, miR-128 mimic, HIC1 vector, or miR-128 mimic plus HIC1 vector. **(E**–**G)** The proliferation, invasion, and apoptosis assays were performed after the transfection. The results are presented as the mean ± S.E. of three independent experiments. *P < 0.05; **P < 0.01; ***P < 0.001.

HIC1 is known to inhibit cell proliferation and invasion and induce apoptosis, so we measured the effects of miR-128-driven repression of HIC1 expression on the proliferation, invasion, and apoptosis of breast cancer cells. First, we measured the effects of miR-128 on cell function and the results indicated that MCF-7 cells overexpressing miR-128 showed increased proliferation, whereas transfecting with miR-128 inhibitors showed the opposite effects (**Figure 5A**). Besides, cells overexpressing miR-128 exhibited promoted cell invasion, whereas cells transfected with anti-miR-128 showed decreased invasion (**Figure 5B**). Analogously, the cells overexpressing miR-128 exhibited decreased apoptosis, whereas cells transfected with anti-miR-128 showed increased apoptosis (**Figure 5C**). Additionally, when MCF-7 cells were co-transfected with HIC1-overexpression plasmids and miR-128 mimics, HIC1 significantly weakened the stimulated effect of miR-128 on cell invasion and proliferation and the inhibitory effect on cell apoptosis (**Figures 5D–G**). In addition, the above experiments were also repeated in T-47D (ER positive) cell line, and we observed consistent results (**Supplemental Figures 3C–E**).

### miR-128-HIC1 Axis Influence BRC Tumorigenesis

**Figure 6:**
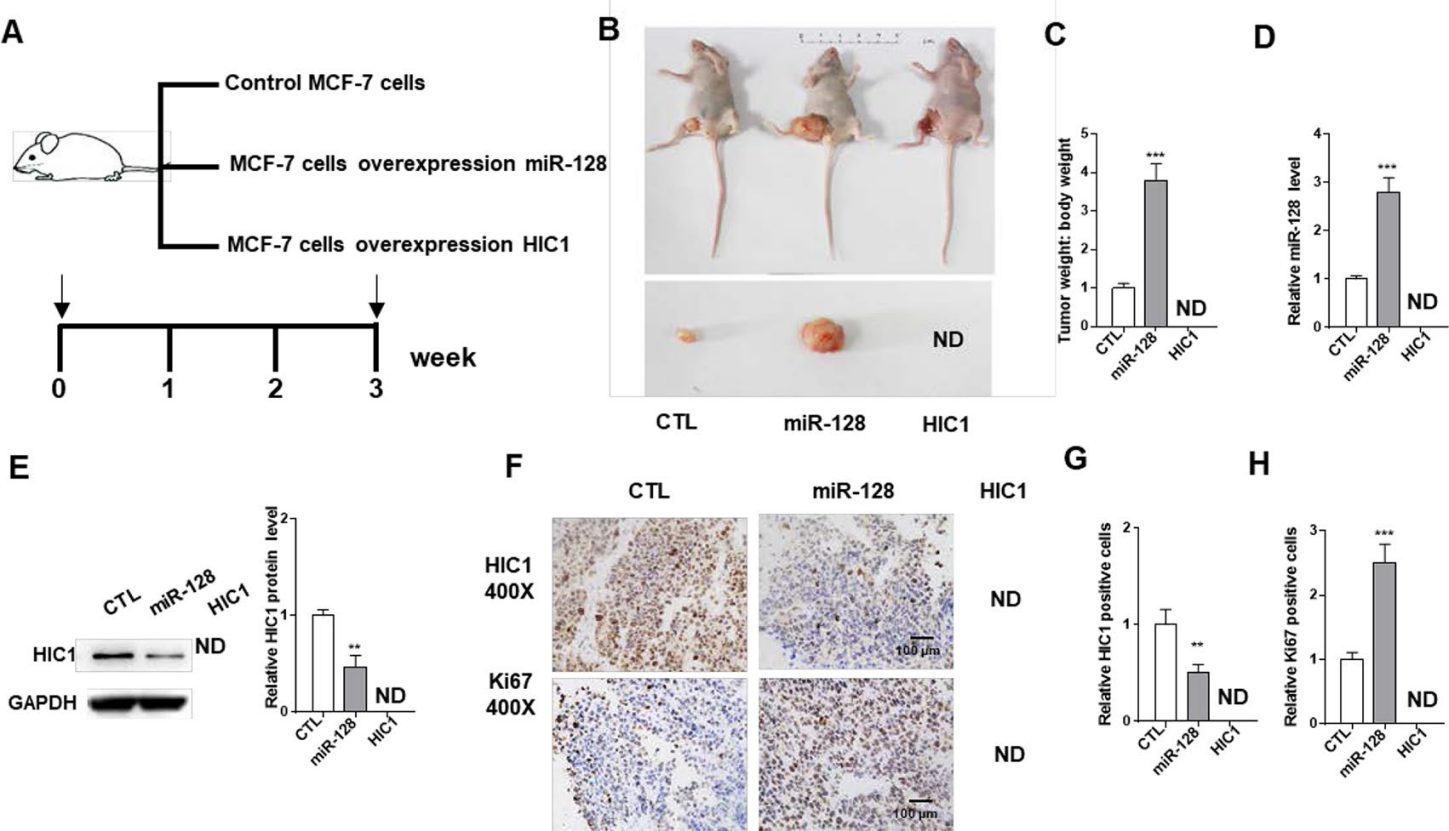
The influence of miR-128 and HIC1 on tumor growth *in vivo*. **(A)** Flow chart of the experimental design. MCF-7 cells were infected with a control lentivirus or a lentivirus to overexpress miR-128, transfected with a HIC1 overexpression plasmids. MCF-7 cells with different treatments were implanted subcutaneously into 6-week-old SCID mice (5 mice/group), and the tumor growth was measured after cell implantation. **(B)** Representative images of tumors and the implanted mice. **(C)** Quantitative analysis of tumor weights. **(D)** Quantitative RT-PCR analysis of miR-128 levels in tumors from implanted mice. **(E)** Western blotting analysis of HIC1 protein levels in the tumors from implanted mice. Left: representative image; right: quantitative analysis. **(F**–**H)** Immunohistochemical staining for HIC1 and Ki-67 in the tumors from implanted mice. F: representative image; G and H: quantitative analysis. The results are presented as the mean ± S.E. of three independent experiments. **P < 0.01; ***P<0.001.

Next, we explored the effects of miR-128-HIC1 axis on xenograft growth in mouse model. MCF-7 cell was infected with a miR-128 overexpression lentivirus. **Supplementary Figure 2** shows the efficient overexpression of miR-128 and suppression of HIC1 in MCF-7 cells. Subsequently, MCF-7 cells (5 × 10^6^ cells per 0.1 mL) were infected with miR-128 lentivirus or transfected with the HIC1 plasmids. Then, the cells were implanted subcutaneously into 6-week-old female SCID mice, and the tumor growth was measured at day 21 after cell implantation (**Figure 6A**). We observed that the tumors in miR-128-overexpressing group showed a faster growth in the sizes and weights than control group (**Figures 6B, C**). Strikingly, we could not find any tumors in HIC1-overexpressing group (**Figures 6B, C**). Then, total RNA and protein were extracted from the tumors and miR-128 and HIC1 levels were measured. After 21 days of tumor growth *in vivo*, tumors from miR-128 overexpression group showed a marked increase in miR-128 expression and displayed decreased HIC1 protein levels compared to control group (**Figures 6D, E**). In addition, immunohistochemical studies showed lower HIC1 expression in miR-128-overexpressing group (**Figures 6F, G**). Finally, Ki-67 immunocytochemistry assay showed that tumors from the miR-128-overexpressing group exhibited increased tumor cell proliferation rate (**Figure 6F, H**). The above results collected *in vivo* were consistent with the findings in *in vitro* assays and firmly validated the biological role of HIC1 and miR-128 in breast tumorigenesis.

## Discussion

In the last few years, with better understanding of breast cancer biology, more and more targeted therapies related to tumorigenesis and progression have been found. However, as the drug resistance and deficient understanding of pathogenesis, the efficacy of new treatments remains limited (Coates et al., 2015). The tumor suppressor gene HIC1, which encodes a transcriptional suppressor, is involved in many cancer processes, such as cell survival, growth, and motility. HIC1 is epigenetically silenced in many human cancers, including breast cancer, prostate cancer, colorectal cancer, liver cancer, and lung cancer (Chen et al., 2005; Cheng et al., 2014), and this is assumed to be ascribed to promoter hypermethylation. However, HIC1 promoter hypermethylation is not only detected in solid tumors but also in normal breast ductal (Zhang et al., 2010), prostate epithelium tissues (Morton et al., 1996), and brain (Rood et al., 2002). These findings suggest that a previously uncharacterized regulatory mechanism rather than hypermethylation is involved in HIC1 repression. In breast cancer, HIC1 deletion had been reported to promote breast cancer progression by activating tumor cell/fibroblast crosstalk (Wang et al., 2018). Moreover, HIC1 could function as tumor suppressor in breast cancer through transcriptional repression of ephrin-A1 (Zhang et al., 2010). In addition, loss of HIC1 in breast cancer cells contributes to stress-induced migration and invasion through β-2 adrenergic receptor (ADRB2) misregulation. Here, we found that miR-128 participates in HIC1 repression in breast cancer cells. Additionally, restoration of HIC1 dramatically weakened the pro-proliferative, pro-invasive, and anti-apoptotic effect of miR128 in breast cancer cells and completely block tumor growth in nude mice. Therefore, HIC1 may be a promising drug target for future breast cancer therapy.

Recently, an important role for miRNA in breast cancer progression has emerged (O’Day and Lal, 2010). While miR-128 had been found upregulated in many types of cancers, such as gastric cancer (Katada et al., 2009), glioma (Pang et al., 2009), and lung cancer (Cai et al., 2017), the expression pattern and pathological function of miR-128 in breast cancer are not clear. In this study, we found that miR-128 was significantly upregulated in breast cancer tissues. The findings indicate that miR-128 might serve as an oncomiR to participate in the pathogenesis of breast cancer. Mechanistic studies showed that miR-128 could directly bind the HIC1 3’-UTR and suppress its expression, promote proliferation and invasion, and inhibit apoptosis of breast cancer cells. More importantly, recovery of HIC1 expression by overexpression plasmids completely reversed miR-128-induced cellular phenotypes, suggesting that targeting HIC1 is a predominant mechanism by which miR-128 exerts its oncogenic function. Moreover, regulation of HIC1 by miR-128 may explain, at least in part, why miR-128 upregulation can increase cell proliferation and invasion and inhibit apoptosis in breast cancer.

In summary, this study reveals a novel axis comprised of HIC1 and miR-128 that may contribute to breast carcinogenesis. The finding may open new avenues for future breast cancer therapies.

## Data Availability Statement

The raw data supporting the conclusions of this manuscript will be made available by the authors, without undue reservation, to any qualified researcher.

## Ethics Statement

The animal study was reviewed and approved by The ethics committee and Institutional Review Board of the First Affiliated Hospital of USTC.

## Author Contributions

YL and XH designed this research. YL and YW carried out most of the experiments, analysed the data, drew the figures and drafted this manuscript. YL collected the BC tissues as well as the paired normal para-carcinoma tissues. YL and XS helped in animal experiment and functional experiment. All authors read and approved the final manuscript.

## Conflict of Interest

The authors declare that the research was conducted in the absence of any commercial or financial relationships that could be construed as a potential conflict of interest.

## Abbreviations

3’UTR, 3’ untranslated region; CCK8, Cell Counting Kit-8; H&E, haematoxylin and eosin staining; miRNA, microRNAs; PTP1B, hypermethylated in cancer 1, HIC1; qRT-PCR, quantitative real-time PCR.
