# Supplementary material for: miR-128 Functions as an OncomiR for the Downregulation of HIC1 in Breast Cancer
Source: Front Pharmacol. 2019 Oct 17;10:1202. doi: 10.3389/fphar.2019.01202 (PMC6811662; doi:10.3389/fphar.2019.01202)
Supplement: Supplementary file 1 [file DataSheet_1.pdf]

Supplementary Table 1. Clinical features of breast cancer patients.

	Age	Gender	Tumor subtype	Pathological Stage
**Case #1**	43	Female	IDC	III
**Case #2**	59	Female	IDC	II
**Case #3**	53	Female	IDC	II
**Case #4**	39	Female	IDC	IIB
**Case #5**	63	Female	IDC	III
**Case #6**	46	Female	IDC	III
**Case #7**	54	Female	IDC	IIA
**Case #8**	45	Female	IDC	IA
**Case #9**	46	Female	IDC	III
**Case #10**	39	Female	IDC	III
**Case #11**	59	Female	IDC	IIB
**Case #12**	62	Female	IDC	II
**Case #13**	54	Female	IDC	II
**Case #14**	57	Female	IDC	III

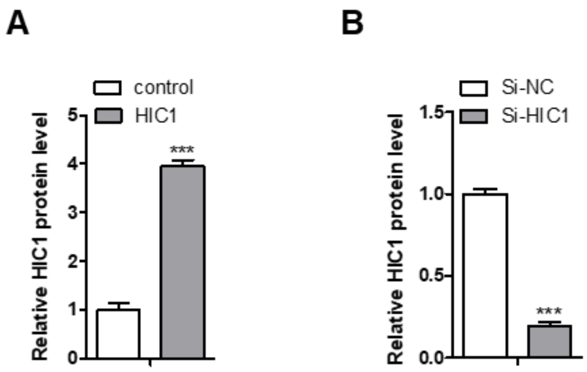

Supplemental Fig.1 Western blot analysis of HIC1 level after the transfection of HIC1 overexpression vector **(A)** and HIC1 siRNA **(B)** in MCF-7. The results are presented as the mean ± S.E. of three independent experiments. *** *P*< 0.001.

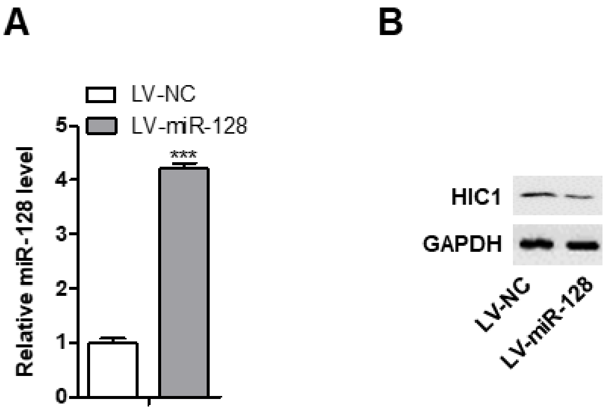

Supplemental Fig.2 (A and B) RT-PCR analysis of miR-128 level (A) and western blot analysis of HIC1 protein levels (B) in MCF-7 cells transfected with control lentiviral (LV-NC) or miR-128 lentiviral (LV-miR-128). The results are presented as the mean ± S.E. of three independent experiments. *** *P*< 0.001.

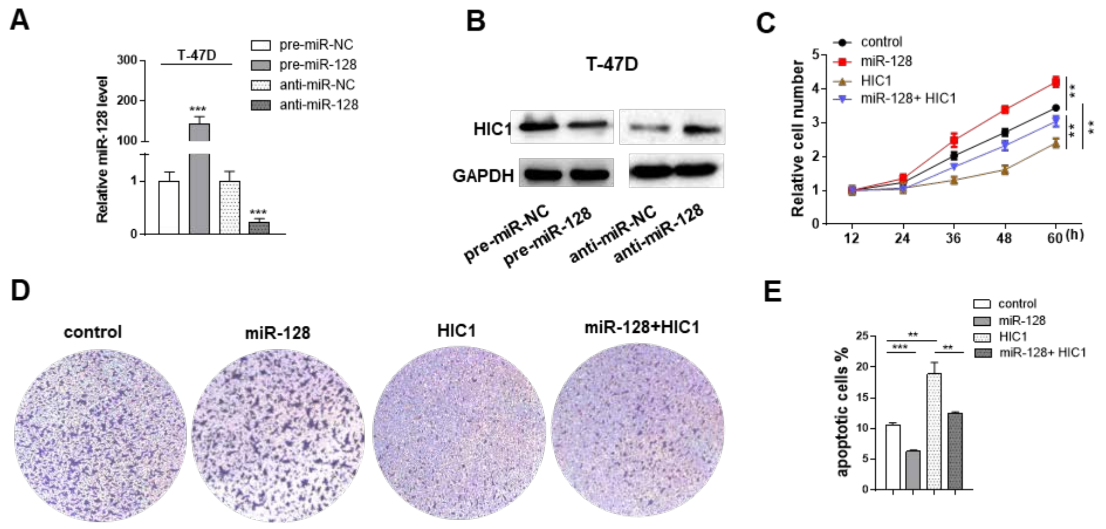

Supplemental Fig.3 miR-128 directly targets HIC1 in T-47D cells. (A) Quantitative RT-PCR analysis of miR-128 levels in T-47D cells transfected with miR-128 mimics, miR-128 inhibitors or scrambled negative control RNA (pre-miR-NC or anti-miR-NC). **(B)** Western blotting analysis of HIC1 protein levels in T-47D cells transfected with miR-128 mimic, miR-128 inhibitor or scrambled negative control RNA. **(C-E)** The proliferation, invasion and apoptosis assays were performed after transfecting with scrambled negative control RNA, miR-128 mimic, HIC1 vector or miR-128 mimic plus HIC1 vector. The results are presented as the mean ± S.E. of three independent experiments. * $P< 0.05$; ** $P< 0.01$; *** $P< 0.001$.
